# A qualitative evidence synthesis to explore healthcare professionals’ experience of prescribing opioids to adults with chronic non-malignant pain

**DOI:** 10.1186/s12875-017-0663-8

**Published:** 2017-11-25

**Authors:** Fran Toye, Kate Seers, Stephanie Tierney, Karen Louise Barker

**Affiliations:** 1 Nuffield Orthopaedic Centre, Oxford University Hospitals NHS Foundation Trust, Windmill Road, Oxford, 0X3 7HE UK; 2 Warwick Research in Nursing, Warwick Medical School, University of Warwick, Coventry, CV4 7AL UK; 3 Nuffield Department of Orthopaedics, Rheumatology and Musculoskeletal Sciences (NDORMS), University of Oxford, Windmill Road, Oxford, 0X3 7LD UK

**Keywords:** Chronic pain, Analgesic, opioid, Qualitative research, Qualitative evidence synthesis, Meta-ethnography

## Abstract

**Background:**

Despite recent guidelines suggesting that patients with chronic non-malignant pain might not benefit, there has been a significant rise in opioid prescription for chronic non-malignant pain. This topic is important because an increasing number of HCPs are prescribing opioids despite very limited evidence for long-term opioid therapy for chronic non-malignant pain outside of end-of-life care. To better understand the challenges of providing effective treatment, we conducted the first qualitative evidence synthesis to explore healthcare professionals’ experience of treating people with chronic non-malignant pain. We report findings that explore healthcare professionals’ experience of prescribing opioids to this group of patients.

**Methods:**

We searched five electronic bibliographic databases (Medline, Embase, CINAHL, PsychINFO, AMED) from inception to November 2015 and screened titles, abstracts and full texts of potential studies. We included studies in English that explored healthcare professionals’ experience of treating adults with chronic non-malignant pain. Two reviewers quality appraised each paper. We used the methods of meta-ethnography developed and refined for large reviews, and the GRADE-CERQual framework to rate confidence in review findings.

**Results:**

We screened 954 abstracts and 184 full texts, and included 77 studies in the full review. 17 of these 77 studies included concepts that explored the experience of prescribing opioids. We abstracted these concepts into 6 overarching themes: (1) Should I, shouldn’t I? (2) Pain is Pain; (3) Walking a fine line; (4) Social guardianship; (5) Moral boundary work; (6) Regulations and guidelines. We used the GRADE-CERQual framework to evaluate confidence in findings. A new overarching concept of ‘ambiguity’ explains the balancing required around the factors taken into account when prescribing opioids. Managing this ambiguity is challenging and these findings can inform healthcare professionals dealing with these decisions.

**Conclusions:**

This conceptual model demonstrates the complexity of making a decision to prescribe opioids to someone with chronic non-malignant pain. Although opioid prescription is underpinned by the therapeutic aim of alleviating pain, this aim may be misplaced. This has implications for education in light of the new regulations for opioid prescription. Findings also demonstrate that the decision is influenced by intra- and interpersonal factors and broader external concerns.

## Background

Population estimates suggest that around 25% of adults around the world suffer with moderate or severe pain [1–5] and for between 6 and 14% of these adults pain can be severe and disabling [5, 6]. Each year over five million people in the UK develop chronic non-malignant pain [7], and healthcare professionals (HCPs) can find it a challenge to effectively treat this pain. For example, they can find it difficult if they are unable to offer the patient a solution [8], or if they have to refuse patients’ requests for a particular test or treatment [9, 10]. Despite recent USA [11] and UK guidelines [12] suggesting that patients with chronic non-malignant pain might not gain benefits, opioids are commonly prescribed for uncontrolled persistent pain, and there has been a significant rise in opioid prescription [13–18]. Survey data suggest that around 12% of UK patients with chronic non-malignant pain are prescribed strong opioids, mainly by GPs [1]. As many as 20% of patients with non-malignant pain symptoms receive an opioid prescription [19].

In order to better understand the challenges of providing effective treatment, we aimed to explore healthcare professionals’ experience of treating patients with chronic non-malignant pain by conducting a qualitative evidence synthesis in this area. A full report of HCPs experience of treating patients with chronic non-malignant pain is being published by the NIHR Journals library [20]. We aimed to focus on the experience of treating chronic pain conditions with no clear attributable biomedical cause. Qualitative evidence synthesis aims to systematically search for and integrate findings in order to increase our understanding of complex processes of care, and thus improve the experience and quality of that care. As part of this review, we found a body of evidence that specifically explored healthcare professionals’ (HCPs) experience of prescribing opioid medication to patients with chronic non-malignant pain. This topic is important because an increasing number of HCPs are prescribing opioids despite very limited evidence for long-term opioid therapy for chronic non-malignant pain outside of end-of-life care [18].

## Methods

We used the methods of meta-ethnography developed, refined and reported in a previous qualitative evidence synthesis of patients’ experience of chronic non-malignant musculoskeletal pain [21]. There are seven stages to meta-ethnography: getting started, deciding what is relevant, reading the studies, determining how studies are related, translating studies into each other, synthesising translations and expressing the synthesis [22].

### Search strategy

We searched five electronic bibliographic databases (Medline, Embase, CINAHL, PsychINFO, AMED) from inception to end of November 2015 using terms adapted from the InterTASC Information Specialists’ Sub-Group (ISSG) Search Filter Resources [23–26]. We combined thesaurus terms from each database with free text terms for qualitative research as a subject, and for pain as a subject (Table 1). Previous research suggests that citation checks, hand searching, grey literature or PhD searches do not necessarily add value to large meta-ethnographies. For example, Toye and colleagues found that 95% of studies were identified in the first three databases searched [21, 27]. FT and KLB screened the titles, abstracts and full text of potential studies for relevance. Any disagreement was discussed and resolved with a third reviewer.

**Table 1:** Example search terms – Medline: (a) qualitative subject headings); (b) qualitative free text terms; (c) pain subject headings; (d) pain free text terms

(I) QUALITATIVE SUBJECT HEADINGS	exp QUALITATIVE RESEARCH exp. INTERVIEWS AS TOPIC exp. FOCUS GROUPS NURSING METHODOLOGY RESEARCH ATTITUDE TO HEALTH
(II) QUALITATIVE FREE TEXT TERMS	Qualitative ADJ5 (theor* OR study OR studies OR research OR analysis) ethno.ti,ab emic OR etic. ti,ab phenomenolog*.ti,ab hermeneutic*.ti,ab heidegger* OR husserl* OR colaizzi* OR giorgi* OR glaser OR strauss OR (van AND kaam*) OR (van AND manen) OR ricoeur OR spiegelberg* OR merleau).ti,ab constant ADJ3 compar*.ti,ab focus ADJ3 group*.ti,ab grounded ADJ3 (theor* OR study OR studies OR research OR analysis).ti,ab narrative ADJ3 analysis.ti,ab discourse ADJ3 analysis.ti,ab (lived OR life) ADJ3 experience*.ti,ab (theoretical OR purposive) ADJ3 sampl*.ti,ab (field ADJ note*) OR (field ADJ record*) OR fieldnote*.ti,ab participant* ADJ3 observ*.ti,ab action ADJ research.ti,ab (digital ADJ record) OR audiorecord* OR taperecord* OR videorecord* OR videotap*).ti,ab (cooperative AND inquir*) OR (co AND operative AND inquir*) OR (co-operative AND inquir*) .ti,ab (semi-structured OR semistructured OR unstructured OR structured) ADJ3 interview*.ti,ab (informal OR in-depth OR indepth OR “in depth”) ADJ3 interview*.ti,ab (“face-to-face” OR “face to face”) ADJ3 interview*.ti,ab “IPA” OR “interpretative phenomenological analysis”.ti,ab “appreciative inquiry”.ti,ab (social AND construct*) OR (postmodern* OR post-structural*) OR (post structural* OR poststructural*) OR (post modern*) OR post-modern* OR feminis*).ti,ab humanistic OR existential OR experiential.ti,ab
(III) PAIN SUBJECT HEADINGS	exp BACK PAIN/OR exp. CHRONIC PAIN/OR exp. LOW BACK PAIN/OR exp. MUSCULOSKELETAL PAIN/OR exp. PAIN/OR exp. PAIN CLINICS/. exp. FIBROMYALGIA/ exp. PAIN MANAGEMENT/
(IV) PAIN FREE TEXT TERMS	(chronic* OR persistent* OR long-stand* OR longstand* OR unexplain* OR un-explain*) fibromyalgia “back ache” OR back-ache OR backache “pain clinic” OR pain-clinic* pain adj5 syndrome*

### Inclusion/exclusion criteria

We included studies written in English that explored HCPS’ experience of treating adults with chronic non-malignant pain. We used a broad search strategy that included ‘PAIN’ as both a thesaurus and free text term in order to encompass all chronic non-malignant pain conditions. We excluded acute pain, head pain, arthritis (including osteoarthritis and rheumatoid arthritis), patient experience and studies where HCP experience could not be disentangled from the experience of others. Our focus was on non-specific chronic non-malignant pain. We did not include HCPs experience of treating patients with arthritis as, after consultation with our advisory group of clinicians and patients, we felt it likely that treating conditions with bio-medically attributable causes would be qualitatively different. At the outset of this study, we had intended to only include HCPs experience of treating chronic non-malignant *musculoskeletal* pain in order to mirror a previous QES of patients’ experience of chronic non-malignant musculoskeletal pain [21]. However our preliminary reading indicated that HCPs experience of treating chronic non-malignant pain was not boundaried to a particular body system, but was a summative experience that cuts across conditions.

### Quality appraisal

Although there are many suggested frameworks, there is no consensus on what makes a qualitative study good [28, 29]. We used three methods of quality appraisal to frame our discussions regarding inclusion: (a) The Critical Appraisal Skills Programme (CASP) questions for appraising qualitative research [30]; (b) constructs from a qualitative study embedded in a previous large meta-ethnography [31]; (c) a global appraisal of whether the study was: a ‘key paper’ (‘conceptually rich and could potentially make an important contribution to the synthesis’); a satisfactory paper; a paper that is irrelevant to the synthesis; a methodologically fatally flawed paper [29]. Two reviewers appraised each paper, and if they were unable to reach an agreement, the study was sent to another reviewer for the final decision. We utilised the GRADE-CERQual framework [32] which aims to help reviewers to assess and describe how much confidence readers can place in review findings. GRADE-CERQual suggest four domains of interest: (1) ‘Methodological limitations’ concern the conduct of the primary study; (2) ‘Relevance’ is the extent to which the primary studies are applicable to the review; (3) ‘Adequacy of data’ is an ‘overall determination of the degree of richness and quantity of data supporting a review finding’; (4) Coherence considers how well the findings are grounded in the primary studies [32].

### Data extraction and synthesis

Two reviewers read each paper to develop a list of the concepts from the primary papers. If they agreed that there was no clear concept then it was not included in the analysis. Translating studies into each other involves comparing concepts in order to sort them into conceptual categories [22]. All reviewers sorted the concepts into categories with shared meaning. Through constantly comparing and discussing these categories they started to see similarities and differences that allowed further abstraction into the final conceptual categories. The final analytic stage, ‘synthesising translations’ involved integrating the conceptual categories into a conceptual framework. We planned to develop a line of argument synthesis, which involves ‘making a whole into something more than the parts alone imply’ [22] (page 28). This is achieved by comparing concepts and developing ‘a grounded theory that puts the similarities and differences between studies into interpretive order’ [22] (page 64).

## Results

**Fig. 1:**
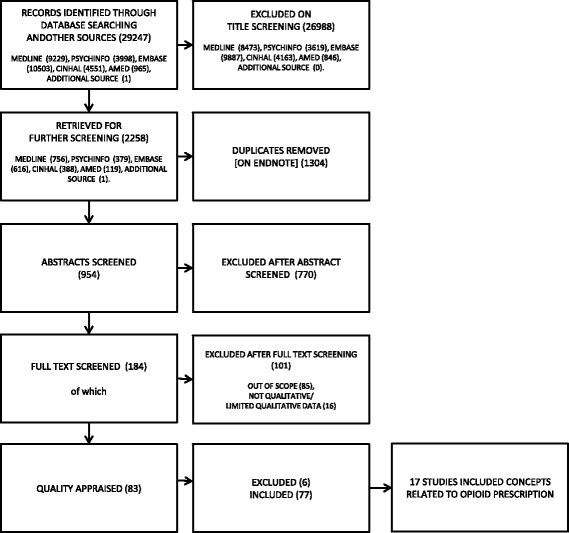
Flow Chart showing results of systematic search: this figure give the records identified, screened, retrieved, appraised and included in the review

We report findings that explored HCPs’ experience of prescribing opioids to patients with chronic non-malignant pain. A full report of HCPs experience of treating patients with chronic non-malignant pain is being published by the NIHR Journals library [20]. The results of the systematic search are shown in Fig. 1. We screened 954 potentially relevant studies and excluded 770 after screening the abstracts. We retrieved 184 full text articles and excluded 101. We excluded 16 studies that were not qualitative or that included limited qualitative data, and 85 studies that we agreed were out of scope (for example, they did not present the HCP voice, or they did not explore the experience of chronic non-malignant pain). Full details of study exclusions are being published elsewhere [20]. Of the 83 studies remaining, we unanimously excluded a further six on the grounds of methodological report. After reading and extracting concepts from the remaining 77 studies, the reviewers identified 17 studies that included concepts specific to the experience of prescribing opioids to patients with chronic non-malignant pain [33–49]. Table 2 gives the author, year, country, participants, data collection, analytic method and aim of the 17 studies included. Findings are drawn from 486 HCPs from different geographic locations; USA (*n* = 10), UK (*n* = 4), Canada (*n* = 2), Spain (*n* = 1) (Table 2).

**Table 2:** Characteristics of 17 studies that included concepts exploring the experience of prescribing opioids to patients with chronic non-malignant pain

AUTHOR YEAR	COUNTRY	PARTICIPANTS	DATA COLLECTION	ANALYSIS	AIM OF STUDY
BALDACCHINO 2010 [33]	SCOTLAND, UK	29 physicians	2 focus groups/19 interviews	Framework analysis	To describe physicians’ attitudes and experience of prescribing opioids for chronic non-cancer pain with a history of substance abuse.
BARRY 2010 [34]	USA	23 physicians	Semi-structured interview	Grounded theory	To identify barriers and facilitators to opioid treatment of chronic non-cancer pain patients
BERG 2009 [35]	USA	16 physicians and ‘assistants’	Semi-structured interview	Thematic analysis	To explore providers’ perceptions of ambiguity, and then to examine their strategies for making diagnostic and treatment decisions to manage chronic pain among patients on methadone maintenance therapy.
BRIONES-VOZMEDIANO 2013 [36]	SPAIN	9 mixed HCPs: (GPs, physicians, physiotherapists, rheumatologists, psychologists, psychiatrist)	Semi-structured interview	Discourse Analysis	To explore experiences of fibromyalgia management, namely diagnostic approach, therapeutic management and the health professional-patient relationship.
ESQUIBEL 2014 [37]	USA	21 family practitioners	In-depth interviews	Immersion-crystallization	To explore the experiences of adults receiving opioid therapy for relief of chronic non-cancer pain and that of their physicians
FONTANA 2008 [38]	USA	9 advanced practice nurses	Semi-structured interview	No specific method identified	To critically examine subjective factors that influence prescribing practices of registered nurses for patients with chronic non-malignant pain.
GOOBERMAN-HILL 2011 [39]	UK	27 general practitioners	Semi-structured interview	Thematic analysis	To explore GPs’ opinions about opioids and decision-making processes when prescribing ‘strong’ opioids for chronic joint pain.
KAASALAINEN 2007 [40]	CANADA	66 mixed HCPs: (Physicians (*n* = 9), registered practical nurses)	Semi-structured interviews/focus groups	Grounded theory	To explore the decision-making process of pain management of physicians and nurses and how their attitudes affect decisions about prescribing and administering pain medications among older adults in long-term care.
KAASALAINEN 2010B [42]	CANADA	53 Mixed HCPs: (15 Registered nurses, 6 registered practical nurses, 4 physicians, 20 unlicensed care practitioners, 2 pharmacists, 2 physiotherapists, 4 administrators)	6 focus groups/interviews	Case-study analysis	To: (1) explore barriers to pain management and those associated with implementing a pain management program in long-term care (LTC); (2) to develop an inter-professional approach to improve pain management in LTC.
KILARU 2014 [41]	USA	61 emergency physicians	Semi-structured interview	Grounded theory	To identify key themes regarding emergency physicians’ definition, awareness, use, and opinions of opioid prescribing guidelines.
KREBS 2014 [43]	USA	14 primary care physicians	Semi-structured interview	Immersion-crystallisation	To understand physicians’ and patients’ perspectives on recommended opioid management practices and to identify potential barriers to and facilitators of guideline-concordant opioid management in primary care.
MCCRORIE 2015 [44]	UK	15 general practitioners	2 focus groups	Grounded theory	To understand the processes which bring about and perpetuate long-term prescribing of opioids for chronic, non-cancer pain.
RUIZ 2010 [45]	USA	19 mixed HCPs: (14 primary care physicians, 5 nurse practitioners)	3 focus groups 9 semi-structured interviews	Grounded theory	To explore the attitudes of primary care clinicians (PCPs) toward chronic non-malignant pain management in older people.
SEAMARK 2013 [46]	UK	22 general practitioners	Interviews/focus groups	Thematic analysis	To describe the factors influencing GPs’ prescribing of strong opioid drugs for chronic non cancer pain.
SIEDLECKI 2014 [47]	USA	48 nurses	Interviews	Grounded theory	To explore and understand nurses’ assessment and decision-making behaviours related to the care of patients with chronic pain in the acute care setting.
SPITZ 2011 [48]	USA	26 Mixed HCPs: (23 physicians, 3 nurse practitioners)	Focus groups	Thematic analysis	To describe primary care providers’ experiences and attitudes towards, as well as perceived barriers and facilitators to prescribing opioids as a treatment for chronic pain among older adults.
STARRELS 2014 [49]	USA	28 physicians	Open ended telephone interview	Grounded theory	To understand primary care providers’ experiences, beliefs and attitudes about using opioid treatment agreements for patients with chronic pain.

All reviewers discussed and organised the concepts into 18 conceptual categories. Only a single concept did not fit into a conceptual categories (Barry 2010: Patient Factors - Cost of Specialty Pain Management [34]). This described how some HCPs felt their patients were concerned about the costs and coverage of specialty pain management and therefore might only be relevant to healthcare systems that are not free at point of delivery. All four reviewers further abstracted the 18 conceptual categories into 6 themes that underpin HCPs’ experience of prescribing opioids to patients with chronic non-malignant pain. A core concept overarching all six themes was a sense of ambiguity surrounding opioid prescribing.

### Confidence in review findings (GRADE -CERQual assessment)

Indicators of confidence in our review findings as recommended in the GRADE -CERQual framework [32] are shown in Table 3. Both reviewers rated all studies as satisfactory. Twelve studies were directly relevant and aimed to explore the experience of opioid prescription [33–35, 37–39, 41, 43, 44, 46, 48, 49]. The remaining five studies were partially relevant and included themes related to opioid prescription. Table 3 shows the number of concepts and the number of studies out of 17 supporting each review finding. We rated our confidence in the review finding as high when it was supported by more than half of the studies. However, there is currently no agreed way of making an assessment of confidence for qualitative synthesis.

**Table 3:** Confidence in review findings – GRADE -CERQual assessment

REVIEW FINDING	METHODOLOGICAL LIMITATIONS (NUMBER OF SATISFACTORY STUDIES)	RELEVANCE (PARTIAL OR DIRECT)	ADEQUACY (NUMBER OF CONCEPTS)	COHERENCE* (NUMBER OF STUDIES OUT OF 17)	OVERALL ASSESSMENT OF CONFIDENCE
SHOULD I, SHOULDN’T I?	ALL	9 DIRECT	19	9 [34–39, 43, 44, 48]	HIGH CONFIDENCE
PAIN IS PAIN	ALL	5 DIRECT, 1 PARTIAL	8	6 [33, 39, 40, 42, 46, 48]	MODERATE CONFIDENCE
WALKING A FINE LINE	ALL	9 DIRECT, 1 PARTIAL	16	8 *[33, 35, 39–41, 45, 46, 48]*	MODERATE CONFIDENCE
SOCIAL GUARDIANSHIP	ALL	10 DIRECT, 1 PARTIAL	17	11 [33–35, 37–39, 41, 43, 45, 46, 48]	HIGH CONFIDENCE
MORAL BOUNDARY WORK	ALL	12 DIRECT, 2 PARTIAL	27	14 [33–35, 37–39, 41, 43–49]	HIGH CONFIDENCE
REGULATIONS AND GUIDELINES	ALL	8 DIRECT	18	8 [34, 35, 38, 39, 41, 43, 48, 49]	MODERATE CONFIDENCE

### Thematic analysis

Our synthesis supports six themes that can help to understand HCPs experience of prescribing opioids to patients with chronic non-malignant pain: (1) Should I, shouldn’t I? (2) Pain is Pain; (3) Walking a fine line; (4) Social guardianship; (5) Moral boundary work; (6) Regulations and guidelines. We have illustrated each theme with examples of narrative from the primary studies.

#### Should I, shouldn’t I?

The theme ‘should I, shouldn’t I?’ describes the sense of uncertainty about when to prescribe opioids, and a feeling of ambiguity about the effects of medication. This feeling was heightened by an observation that, in similar circumstances, other HCPs seemed to make different clinical decisions:

When we’re practicing alongside other people who have come to completely different conclusions, it really makes you think … have I been making the wrong decisions; did we get different information and come to different conclusions? Do we have different values that underlie our decision-making? [35] (physician, USA)

HPCs found it harder to approach analgesic prescribing where the disease aetiology was unknown, for example, in fibromyalgia.

Because you don’t really know what’s happening there. The aetiology of the disease is not really known and you have few means of knowing what you’re doing. You’re treating the pain and you don’t know why there is no response [36] (rheumatologist, Spain)

They are in pain, you give them something for the pain and: ‘it doesn’t do me any good … it relieved the pain a little but the pain has come back’ … . No matter what you give them, the pain doesn’t go away [36] (GP, Spain)

There was a sense that clinical education did not prepare HCPs adequately for these decisions.

We took an advanced pharm[acology] class, and we discussed it in one lecture, but that was it. Isn't that ridiculous considering how many people we see in pain? [38] (practise nurse, USA)

Uncertainty was also compounded by the sense that specialist referrals were either restricted or unproductive. Some HCPs felt unsupported in managing the most difficult cases and explored the possibility of more specialist services: for example, for patients with chronic pain and substance abuse:

Often I find that they are not accomplishing any more than I was and [patients] are often sent back to me with them [pain specialists] essentially saying, ‘we did our best.’ It’s very frustrating, because if they were easy patients they wouldn’t have been seeing them … they wouldn’t have been referred … I would love for there to be a separate clinic where I could refer patients for management of their chronic pain and substance abuse simultaneously. Kind of take me out of the picture [34] (physician, USA)

#### Pain is pain

The theme ‘pain is pain’ is underpinned by the ideal that if a person is in pain then the primary aim of the HCP should be to relieve pain. Some felt that even addiction should not be a barrier to opioid prescription and described the stigma against patients with addiction:

I had a guy last week who'd been stabbed and he'd been in ITU … and he had to discharge himself because they wouldn't give him any pain control … he wasn't even getting his prescribed dose of methadone he was getting under dosed for his addiction and his pain control … there's a protocol … but they choose not to know about it and it's just pure stigma. [33] (physician, UK)

At the end of the day, if someone’s got chronic pain it doesn’t matter if they’re addicted to painkillers if it sorts out their quality of life [39] (GP, UK)

However, although in *theory* ‘pain is pain’, HCPs described factors that would make them less likely to prescribe opioids in practice for chronic non-malignant pain. For example, whereas for malignant pain the aim might be to achieve complete pain relief, in non-malignant pain the HPC would need to consider the balance of risks and benefits of long term opioid prescription over time:

I don’t regard them [malignant and non-malignant pain] the same … with [malignant pain] … your aim always is to get complete relief of pain … over a finite period of time. For chronic pain … you’ve got to weigh up … the potential side effects… I think there has to be an acceptance that you are not necessarily going to get them pain free because they’ve got the rest of their lives to live as well … so your two end points are different [46] (GP, UK)

Some HCPs queried whether enough attention was given to chronic non-malignant pain when the focus was mainly on pain control in palliative patients.

The only people in my practice that I prescribe [opioids] to would be people who are palliative … . [on the other hand] we tend to focus too much on pain control for palliation as opposed to just everyday clients. Certainly nobody wants to die in pain, but nobody wants to live in pain either [40] (physician, Canada)

#### Walking a fine line

The theme ‘walking a fine line’ describes the need for HCPs to carefully balance the benefits and adverse effects of opioids. On the one hand, emphasising adverse effects might lead to unnecessary pain; on the other hand, emphasis on pain control might lead to harm or abuse. Some HCPs felt that treating pain should take priority over the risk of opioid misuse when making prescribing decisions.

I mean there are two mistakes you make. You can make the mistake of under treating or of giving medicines that end up being sold or used for unintended purposes. You’re going to make errors both ways, and I think it’s generally better to risk opiates being misused versus not treating someone’s pain [35] (physician, USA)

There were additional concerns about prescribing opioids to older adults because of the potential severity and impact of adverse effects. In theory a person’s age should not affect decisions, but in practise, there was a sense that it does.

But there are safety issues, and at the end of the day if they came to grief and fell over, fell down the stairs and broke something, died, then, you know, you’d feel guilty about giving them adequate pain relief in your view, but excessive side effects, drowsiness, what have you, that contributed to some major event on their part. So we walk at a fine line sometimes between giving adequate pain relief and giving safe treatment [39] (GP, UK)

Although HCPs felt that opioids should be used to manage older peoples’ pain, they also discussed how the risks and benefits of prescribing opioids would require assessment on an individual basis.

Older people metabolize medication differently than younger people, so you don’t want to give them medication that’s going to impair their ability to function … A lot of them drive even though they may be even much older, so you don’t want falls. You don’t want automobile accidents. You don’t want injuries. You don’t want to interfere with their ability to make judgments and so on, so I don’t like using opioids in elderly people at all … *[however]* my feeling is that they should be used, but we need to, again, look at each person individually, and then determine which person benefits from opioid therapy [45] (HPC not stated, USA)

#### Social guardianship

The theme ‘social guardianship’ describes a culture hostile to opioid use and the professional taboo of prescribing opioids. HCPs compared their own prescribing practise to their colleagues and were concerned over being judged by their peers. Some felt that they had a personal responsibility to protect society from the consequences of opioid misuse and viewed patients with suspicion, particularly if they requested opioids. To protect society, some implemented strategies to control patients’ behaviour (for example: bottle checks, opioid contracts, background checks). HCPs also described concerns over diversion of opioid prescriptions to others.

I am a naysayer on opiates … too much of my day is spent policing how many [opioids] have been prescribed and how many times a patient is a return patient and how often they visited requesting opiate prescriptions [41] (emergency physician, USA)

If you prescribe to a population where you think diversion is going on, you definitely have a responsibility. I also worry about who is getting the drug, is it my son? I mean, we are members of society after all… . I think it is okay to go into a relationship with some mistrust. It is survival in the business we are in [38] (advanced practice nurse, USA)

Some HCPs discussed indicators of potential abuse (for example: lost prescriptions, early requests for medication, frequent attendance), although acknowledged that these might actually indicate poorly managed pain.

If people are taking it genuinely for pain they tend to stick to the prescribed dosage … addicts tend to be the ones who are always ordering early … you don't lose your tablets if you are … getting great benefit from them for pain [33] (physician, UK)

The concern would be is this pain real, or is it just put on to obtain opioid? … I mean, an assessment of the pain and whether I think it’s genuine or not. I think it’s very difficult; it’s something I’m currently dealing with at the moment, and not very successfully [46] (GP, UK)

#### Moral boundary work

The theme moral boundary work is underpinned by the clinical work of deciding whose pain is ‘real’ and thus who should be prescribed opioids. HCPs described ideal patients (for example, those that made appropriate demands, took advice and did not cause trouble) and ‘difficult’ patients (the demanding, non-adherent and trouble-making). They made judgments based on non-clinical factors about whether or not a patient was ‘legitimate’.

For those patients that have a legitimate reason for wanting to take it and if I can trust them—that they are not selling, they’re not abusing, and most of these are older patients of mine. They never request early refills, they don’t go to the [emergency room] in between visits to get them—there’s no need for me to do periodic drug screenings and so forth (primary care physician, USA) [43]

Pain with no biomedical diagnosis, vague symptoms or dissonance between a patient’s report of pain and professional observation could trigger suspicion.

A lot of patients you can tell … that they really need it … based on their underlying pathology, for example, a patient who has a cancer or a real anatomic foundation for it … now this is not 100% reliable, but you have to count on more observation, combined with other clinical data. So after 2 or 3 visits, you pretty much know who is abusing and who is not [43] (primary care physician, USA)

I think for patients who have chronic pain it’s more challenging and I think that’s the place where I’m constantly rethinking my practice… You’re always on the fence: am I doing the right thing for my patient? [41] (Emergency physician, USA)

Non-clinical moral judgments or *gut-feelings* contributed to prescribing decisions. HCPs recalled episodes when they had made a mistake by trusting ‘the wrong’ patient. Over time they described how they had become better at making the *right* decision.

The way I behave now prescribing for everything is a sort of rather woolly, nebulous product of everything I’ve done… You just pick it up over the years, so I’m sure I’ve been moulded by the successes and the failures which have come my way … we all learn on the hoof, don’t we?’ … I think everybody’s fingers get burnt with people who you give the opioids to with a more trusting attitude than maybe you should have [46] (GP, UK)

I’ve had trust in people, and it’s been betrayed … I find I’m not always that great a judge of who to trust and who not to trust… . I think people feel like they’ve been violated, you know, cheated, like they’ve been taken advantage of. I feel some of that, too. Ultimately you feel you’ve made a poor judgment, and you get mad at yourself … My impression was that he had a true ankle problem. Then you find out it was all lies … you’re allowed to make mistakes [35] (physician, USA)

However, some sensed the dangers of judging a book by its cover and acknowledged that basing clinical decisions on their gut feeling was not fair or accurate.

There’s a disconnect … even if it’s the sweetest little 85-year-old woman who looks like your grandmother, versus, you know, some guy from the ghetto wearing his pants down at his knees … it shouldn’t really matter [49] (physicians, USA)

#### Regulations and guidelines

The theme regulations and guidelines depict HCPs’ views about external regulation of opioid prescription (specifically guidelines, opioid agreements and drug screening). Some described a negative view of prescribing guidelines and felt that they interfered with professional autonomy; an example of the ‘legislature practising medicine without a licence’ [41].

You’re there to help them and they can tell you their deepest, darkest secrets, but yet you’re policing them… . I’m not a big drug screen person, to be honest, because I like to see the person as a person. I’m not clouded by all this other stuff … You can’t do your job when you are thinking about these things [43] (primary care physician, USA)

There could be negative implications to that if patients are actually leaving the emergency department because of the way they interpret that [regulations] poster … there’s potential that sick patients could actually leave your emergency department when they need help [41] (emergency physician, USA)

HCPs described fears legislative reprimand.

My name is on that bottle. If they lose it and someone else takes it and they die, who do you think they are going to come to? What if it is a kid who takes it just for fun—my name is on that, not yours. … I had a patient die. He took the entire bottle, and the police came to see me because they found him dead with the empty bottle with my name on it, and I say to patients now, ‘I am only going to give you a small amount, because I don't want you found dead with my name on your bottle.’ [38] (Advanced practice nurse, USA)

Others described a negative view of opioid prescribing agreements and drug screening as striking a blow at the heart of a patient-clinician relationship by creating mistrust and hostility.

It can really strike a major blow to trust in the doctor patient relationship when you ask someone to sign a piece of paper … A huge power play on the part of the doctor … if there is already mistrust between the patient and the doctor, it could heighten that mistrust … .It takes work on the provider’s part, to make it an alliance-building instrument instead of a punitive contract [49] (physician, USA)

Some HPCs described a more positive attitude to regulation. For example, opioid agreements could be useful in establishing boundaries and opening up honest discussion.

I think it improves the care, because you are able to then have more open and frank discussions around their pain … . and [about] other things going on in their life … In the best of circumstances it actually will make for a deeper more trusting relationship [49] (physician, USA)

Some used guidelines as leverage or to justify decisions and thus help them to deal with ‘challenging’ patients.

I tell them this is standard protocol. I’m not singling you out. I’m not picking on you. I’m not treating you like an addict. This would happen to anybody. If you take our chronic pain meds long enough, anybody will become physically dependent on them [43] (primary care physician, USA)

[An agreement] gives me leverage or comfort in discontinuing the medication if the patient violates the agreement, because we’ve kind of laid it out from the beginning that those behaviors were not okay… . it made my life a little easier, but I’m not sure it did the patients a giant service [49] (physician, USA)

### Summary and conceptual framework

We developed six themes that help us to understand HCPs experience of prescribing opioids to patients with chronic non-malignant pain:

- *‘Should I, shouldn’t I?’* demonstrates feelings of ambiguity about describing opioids for chronic non-malignant pain; ‘*Pain is pain’* demonstrates that although the ideal aim is to alleviate pain, in practice there are reasons why a HCP might not prescribe opioids to people with chronic non-malignant pain; *‘Walking a fine line’* describes the need to balance the benefits and adverse effects of opioids; *‘Social guardianship’* describes a culture hostile to opioid use and a feeling of personal responsibility to police and protect society; ‘*Moral boundary work’* describes the work of deciding whose pain is ‘real’; *‘Regulations and guidelines’ describes* ambivalence towards external regulation and guidelines.

**Fig. 2:**
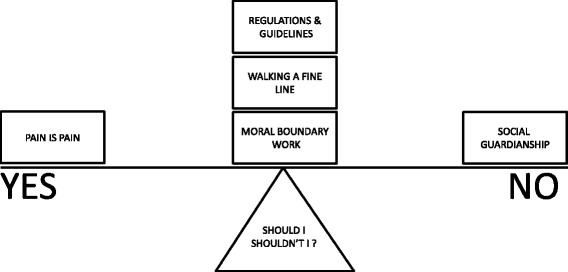
Conceptual framework: this figure illustrates the conceptual framework that demonstrates the complex decision ‘Should I shouldn’t I?’ prescribe opioids for chronic non-malignant pain. The decision is underpinned by the need to decipher ambiguity. ‘Social guardianship’ tips the balance against a decision to prescribe, whereas a sense that ‘pain is pain’ tips the balance towards prescribing. However the decision is not straightforward. The influence of the themes, ‘moral boundary work’, ‘walking a fine line’ and ‘regulations and guidelines’ are not clear-cut and add complexity to the decision

Our conceptual model hinges on the HCPs need to decipher the ambiguity surrounding opioid prescription for chronic non-malignant pain and (*Should I, shouldn’t I*?) (Fig. 2). Social suspicion and hostility toward opioids (*social guardianship*) will tip the balance for prescribing towards the negative, whereas the pre-eminence of pain (*pain is pain*) might tip the balance toward prescribing. The decision is not clear-cut. Firstly, HCPs might make non-clinical judgments about the person (*moral boundary work*), thirdly, they must determine the balance of positive and adverse effects for each individual (*walking a fine line*) and finally there is a sense of professional ambivalence towards prescribing guidelines (*regulations and guidelines*). This conceptual model demonstrates the complexity of making a decision to prescribe opioids to someone with non-malignant pain. It also demonstrates that the decision is influenced by intra- and interpersonal factors and broader external concerns.

## Discussion

This exploration of HCPs experience of prescribing opioids is embedded within a wider qualitative evidence synthesis that aims to further understand healthcare professionals’ experience of treating patients with chronic non-malignant pain [20]. The findings of qualitative research are an interpretation of data. The centrality of interpretation to qualitative research is also its strength; it aims to challenge and develop ideas rather than test theories. In collaborative projects *individual* interpretations enter into a dialectic process where they are challenged and modified. Our model is based on a rigorous collaborative process that included the research team and an advisory group that included patients and clinicians. The innovation of our study is to identify concepts that explore HCPs’ experience of prescribing opioids and to provide a conceptual framework to help us to understand its complexity. This topic is important because an increasing number of HCPs are prescribing opioids despite very limited evidence for long-term opioid therapy for chronic non-malignant pain outside of end-of-life care [18]. In light of recent USA Guidelines [11] and UK [12] resources, our conceptual model can help us to try and unpick why HCPs prescribe opioids for chronic non-malignant pain. Understanding that this process is complex and that guidelines are only one aspect of the process can help suggest how we can address changes to opioid prescribing. Ten out of the 17 studies explored experiences in the USA where the recently introduced bill to congress on opioid abuse prevention and treatment suggests a move towards tighter controls (https://www.congress.gov/bill/115th-congress/house-bill/993/text). Their guidelines [11] conclude “evidence on long-term opioid therapy for chronic pain outside of end-of-life care remains limited, with insufficient evidence to determine long-term benefits versus no opioid therapy … and extensive evidence shows the possible harms of opioids”. Similarly, a UK resource to support the prescribing of opioids [12] stated “there is little evidence that they [opioids] are helpful for long term pain.” There is thus a drive to move away from prescribing opioids for chronic non-malignant pain.

Our conceptual model demonstrates that opioid prescription is underpinned by the therapeutic aim of alleviating pain. HCPs reported a professional duty to get rid of pain (‘*pain is pain’).* However, recent guidelines are clear that patients who do not achieve useful pain relief from opioids within 2–4 weeks are unlikely to gain benefit in the long term [11, 12]. It might be anticipated that reported inefficacy of opioids for chronic non-malignant pain would be a significant reason for *not* prescribing. However, our findings do not indicate that the limited efficacy of opioids for chronic non-malignant pain is a barrier to prescribing. This has clear implications for education, policy and practice. That professionals expressed the importance of alleviating pain suggests the necessity of an intervention that discontinues opioids if they show no benefit. A study is underway in the UK that is testing an intervention to reduce opioid use and provide other support for people with chronic non-malignant pain. The primary studies in this review were published between 2007 and 2015, thus pre-dating the recent guidelines [11, 12]. It is therefore important to consider any potential delay, inherent in systematic reviews, between publication and impact on practice.

Findings demonstrate that although some HCPs were aware of the adverse effects of opioids (‘walking a fine line’), they remained concerned that deciding not to prescribe would lead to unnecessary suffering. In addition, although HCPs discuss potential harm from opioids, the focus was on preventing abuse and addiction. It could be argued that the elephant in the room is the increasing death rate from opioid-related prescription [50, 51]. Between 2003 and 2013, the age-adjusted drug poisoning death rate involving opioid analgesics in the USA increased from 2.9 to 5.1 deaths per 100,000 population [51]. In a Canadian study (1997–201), 1.8% of those prescribed opioids escalated to high dose therapy and 2% died of opioid-related causes while on treatment [50]. Our findings also support a feeling of ambiguity about the efficacy of opioid medication for people with chronic non-malignant pain (‘Should I, shouldn’t I?’), which indicates a gap in knowledge to be filled.

We found that a strong antagonist for prescribing opioids was a feeling of *personal* responsibility to police and protect society from opioid misuse (‘social guardianship’). This was aligned with the ‘*Moral boundary work’* of determining who had ‘real’ pain and who might potentially be playing the system. A qualitative evidence synthesis of patients’ experience of living with chronic non-malignant pain [21] has shown they struggle to prove their legitimacy and experience their pain as adversarial and contested; patients with chronic non-malignant pain often experience the shame and stigma of not fitting the medical model and struggle to *prove* their credibility to others [21]. An environment of mistrust and suspicion is likely to add to this negative experience of healthcare. A more positive and collaborative frame for making the decision to prescribe, or not, might be useful. For example, emphasising the limited efficacy and serious adverse effects of opioids [11, 12].

Finally, our findings demonstrate ambivalence towards the external regulation of opioid prescription. There was a sense that regulation limited professional autonomy and that there should be freedom to prescribe. Some felt that regulation could lead to mistrust and hostility. Others felt that opioid agreements could help to open up honest discussion. Others used them to justify difficult or unpopular decisions. Our search strategy focused generically on chronic non-malignant pain. Further research might usefully focus on specific diagnoses, such as osteoarthritis or rheumatoid arthritis, in order to explore potential similarities and difference in HCP experience of treating these conditions.

## Conclusion

The innovation of this study is to provide a synthesis of qualitative research that helps to explain the challenges involved with prescribing opioids to patients with chronic non-malignant pain. We demonstrate that syntheses of qualitative research can help us to understand complex processes of care and for the first time uncover a new overarching concept of “ambiguity” that explains the balancing required around many of the factors HCPs take into account when prescribing opioids for chronic non-malignant pain. Our findings can inform HCPs dealing with these difficult decisions. Findings demonstrate that the decision is influenced by intra- and interpersonal factors and broader external concerns. Although opioid prescription is underpinned by the therapeutic aim of alleviating pain, this aim may be misplaced. In view of the rise of opioid prescription and recent guidance, further research to explore HCPs’ experience and view of guidelines would be useful. Research to explore patients’ experience of deciding to take opioids for chronic non-malignant pain is also timely. This would help us to understand the motivations and experiences of those with chronic non-malignant pain, and the HCPs trying to manage that pain.

## References

[1] Breivika H (2006). Survey of chronic pain in Europe: prevalence, impact on daily life, and treatment. Eur J Pain.

[2] Elliott AM (1999). The epidemiology of chronic pain in the community. Lancet.

[3] König HH (2009). Comparison of population health status in six European countries: results of a representative survey using the EQ-5D questionnaire. Med Care.

[4] Covinsky KE, Lindquist K, Dunlop DD, Yelin E (2009). Pain, functional limitations, and aging. J Am Geriatr Soc.

[5] Croft P, Peat G, Van-der-Windt D (2010). Primary care research and musculoskeletal medicine. Primary Health Care Res Dev.

[6] Smith B (2001). The impact of chronic pain in the community. Fam Pract.

[7] Department-of-Health, Pain breaking through the barrier*.* Chief Medical Officer’s report, 2009. 150th annual report of the Chief Medical Officer.

[8] Toye F, Jenkins S (2015). ‘It makes you think’ – exploring the impact of qualitative films on pain clinicians. Br J Pain.

[9] Parsons S (2007). The influence of patients' and primary care practitioners' beliefs and expectations about chronic musculoskeletal pain on the process of care: a systematic review of qualitative studies. Clin J Pain.

[10] Breen A (2007). "you feel so hopeless": a qualitative study of GP management of acute back pain. Eur J Pain.

[11] Dowell D, TM. Haegerich, and R. Chou, CDC (Centers for Disease Control and Prevention) Guideline for Prescribing Opioids for Chronic Pain — United States, 2016. Morb. Mortal. Wkly. Rep, 2016. Report 65, March 15: p. 1–49. 10.15585/mmwr.rr6501e1 26987082

[12] Royal-College-of-Anaesthetists, Opioids Aware: A resource for patients and healthcare professionals to support prescribing of opioid medicines for pain*.* 2016(8th November 2016).

[13] Ruscitto A, Smith BH, Guthrie B (2015). Changes in opioid and other analgesic use 1995–2010: repeated cross-sectional analysis of dispensed prescribing for a large geographical population in Scotland. Eur J Pain.

[14] Zin CS, Chen L-C, Knaggs RD (2014). Changes in trends and pattern of strong opioid prescribing in primary care. Eur J Pain.

[15] Levy B (2015). Trends in opioid analgesic-prescribing rates by specialty, U.S., 2007–2012. Am J Prev Med.

[16] Paulozzi LJ, Mack KA, Hockenberry JM (2014). Vital signs: variation among states in prescribing of opioid pain relievers and benzodiazepines—United States, 2012. MMWR Morb Mortal Wkly Rep..

[17] Fredheim O (2009). Prescription pattern of codeine for non-malignant pain: a pharmacoepidemiological study from the Norwegian prescription database. Acta Anaesthesiol Scand.

[18] Chou R, et al. The Effectiveness and Risks of Long-Term Opioid Treatment of Chronic Pain. The Evidence Report/Technology Assessment, Number 218, 2014. Prepared by the Pacific Northwest Evidence-based Practice Center under Contract No. 290–2012-00014-I (Agency for Healthcare Research and Quality (AHRQ) Publication No. 14-E005-EF). https://effectivehealthcare.ahrq.gov/topics/chronic-pain-opioid-treatment/research/.

[19] Daubresse M (2013). Ambulatory diagnosis and treatment of nonmalignant pain in the United States, 2000-2010. Med Care.

[20] Toye F, Seers K, Barker K. 2015–17. www.journalslibrary.nihr.ac.uk/programmes/hsdr/1419807/#/.

[21] Toye F (2013). A meta-ethnography of patients' experiences of chronic non-malignant musculoskeletal pain. Health Services and Delivery Res.

[22] Noblit G, Hare R. Meta-ethnography: Synthesising Qualitative Studies. Qualitative research methods series 11. 1988. California: Sage Publications.

[23] Wong SS, Wilczynski NL, Haynes RB (2004). Developing optimal search strategies for detecting clinically relevant qualitative studies in MEDLINE. Medinfo.

[24] Wilczynski NL, Marks S, Haynes RB (2007). Search strategies for identifying qualitative studies in CINAHL. Qual Health Res.

[25] McKibbon KA, Wilczynski NL, Haynes RB (2006). Developing optimal search strategies for retrieving qualitative studies in PsycINFO. Eval Health Prof.

[26] Walters LA, Wilczynski NL, Haynes RB (2006). Hedges team., Developing optimal search strategies for retrieving clinically relevant qualitative studies in EMBASE. Qual Health Res.

[27] Toye F, et al. Meta-ethnography 25 years on: challenges and insights for synthesising a large number of qualitative studies. BMC Med Res Methodol. 2014:**14**(80). 10.1186/1471-2288-14-80 PMC4127190 24951054

[28] Campbell R, et al. Evaluating meta-ethnography: systematic analysis and synthesis of qualitative research. Health Technol Assess. 2011;15(43) 10.3310/hta15430 22176717

[29] Dixon-Woods M (2007). Appraising qualitative research for inclusion in systematic reviews: a quantitative and qualitative comparison of three methods. J Health Serv Res Policy.

[30] CASP. Critical Appraisal Skills Programme: making sense of evidence about clinical effectiveness: 10 questions to help you make sense of qualitative research. 2010. http://www.casp-uk.net/casp-tools-checklists.

[31] Toye F (2013). Trying to pin down jelly' - exploring intuitive processes in quality assessment for meta-ethnography. BMC Med Res Methodol.

[32] Lewin S (2016). Using qualitative evidence in decision making for health and social interventions: an approach to assess confidence in findings from qualitative evidence syntheses (GRADE-CERQual). PLoS Med.

[33] Baldacchino A (2010). Guilty until proven innocent: a qualitative study of the management of chronic non-cancer pain among patients with a history of substance abuse. Addict Behav.

[34] Barry T (2010). Opioids, chronic pain, and addiction in primary care. J Pain.

[35] Berg M (2009). Providers' experiences treating chronic pain among opioid-dependent drug users. J Gen Intern Med.

[36] Briones-Vozmediano E (2013). Patients' and professionals' views on managing fibromyalgia. Pain Res Manag.

[37] Esquibel AY, Borkan J (2014). Doctors and patients in pain: conflict and collaboration in opioid prescription in primary care. Pain.

[38] Fontana JS (2008). The social and political forces affecting prescribing practices for chronic pain. J Prof Nurs.

[39] Gooberman-Hill R (2011). Professional experience guides opioid prescribing for chronic joint pain in primary care. Fam Pract.

[40] Kaasalainen S (2007). Pain management decision making among long-term care physicians and nurses. West J Nurs Res.

[41] Kilaru S (2014). How do physicians adopt and apply opioid prescription guidelines in the emergency department? A qualitative study. Ann Emerg Med.

[42] Kaasalainen S (2010). An action-based approach to improving pain management in long-term care. Can J Aging.

[43] Krebs E (2014). Barriers to guideline-concordant opioid management in primary care—a qualitative study. J Pain.

[44] McCrorie C (2015). Understanding long-term opioid prescribing for non-cancer pain in primary care: a qualitative study. BMC Fam Pract.

[45] Ruiz JG (2010). Primary care management of chronic nonmalignant pain in veterans: a qualitative study. Educ Gerontol.

[46] Seamark D, et al. GPs prescribing of strong opioid drugs for patients with chronic non-cancer pain: a qualitative study. Br J Gen Pract. 2013;63(617) 10.3399/bjgp13X675403 PMC3839391 24351498

[47] Siedlecki L (2014). Exploring how bedside nurses care for patients with chronic pain: a grounded theory study. Pain Manag Nurs.

[48] Spitz A (2011). Primary care providers' perspective on prescribing opioids to older adults with chronic non-cancer pain: a qualitative study. BMC Geriatr.

[49] Starrels L (2014). It made my life a little easier: primary care providers' beliefs and attitudes about using opioid treatment agreements. J Opioid Manag.

[50] Kaplovitch E (2015). Sex differences in dose escalation and overdose death during chronic opioid therapy: a population-based cohort study. PLoS One.

[51] National-Center-for-Health-Statistics, Health, United States, 2014: With Special Feature on Adults Aged 55–64. Hyattsville: 2015. 26086064

